# The role of imaging in rheumatoid arthritis

**DOI:** 10.4102/sajr.v22i1.1316

**Published:** 2018-07-11

**Authors:** Kgomotso Kgoebane, Mahmood M.T.M. Ally, Martha C. Duim-Beytell, Farhana E. Suleman

**Affiliations:** 1 Department of Radiology, University of Pretoria, South Africa; 2 Department of Internal Medicine, University of Pretoria, South Africa; 3 Department of Radiology, University of Pretoria, South Africa

## Abstract

Conventional radiographs of the hands and feet have traditionally been used in the diagnosis, management and monitoring of patients with rheumatoid arthritis (RA). However, they are not sensitive enough to detect changes early in the disease process. Erosions may only be visible up to two years after the onset of disease, and soft tissue involvement may not be detected at all. Early diagnosis can also be made challenging as markers such as erythrocyte sedimentation rate and C-reactive protein may be normal in up to 20% – 25% of cases. The latest classification criteria (American College of Rheumatology/European League Against Rheumatism [ACR/EULAR] Rheumatoid Arthritis Classification criteria 2010), often used to diagnose RA, incorporate the role of ultrasound and magnetic resonance imaging detection of synovitis, enabling earlier diagnosis and correct classification of patients. This article looks at the role of the various imaging modalities used in the diagnosis and management of RA.

## Introduction

Rheumatoid arthritis (RA) is a chronic auto-immune disease which is characterised by persistent inflammation and joint damage. Early diagnosis provides a window of opportunity for cost-effective therapeutic intervention. The earlier patients are diagnosed and treated, the better the outcome. Clinical and laboratory assessment of RA remains the cornerstone of diagnosis and monitoring of response to treatment. Early diagnosis can be challenging as the serological and conventional radiological characteristics are often absent. In patients with active disease, acute-phase response laboratory tests such as erythrocyte sedimentation rate (ESR) or C-reactive protein (CRP) may be normal in up to 20% – 25% of cases.^1^ Conventional radiographs of the hand and feet tend to show presence of erosions as late as one to two years after onset of the disease.^2^ The latest classification criteria (American College of Rheumatology/European League Against Rheumatism [ACR/EULAR] Rheumatoid Arthritis Classification criteria 2010) (see Box 1),^3^ often used to diagnose RA, incorporate the role of ultrasound (US) and magnetic resonance imaging (MRI) detection of synovitis in the criteria, enabling earlier diagnosis and correct classification of patients. Studies have outlined patients clinically being assessed as having undifferentiated arthritis, but following US or MRI, classified as RA, impacting on their management.^4^ With the ground-breaking advances made in the management of RA, optimal treatment mandates treating to a target of at least low disease activity. Ongoing disease activity is associated with increased morbidity and premature mortality. Newer imaging applications have an important role to play in early diagnosis, monitoring response and identifying poor prognostic factors.^5^

Box 1 Diagnostic criteria for rheumatoid arthritis: 2010 American College of Rheumatology/European League Against Rheumatism Rheumatoid Arthritis Classification Criteria.

Criteria are based on the confirmed presence of joint synovitis in at least one joint, and absence of another diagnosis that explains synovitis and achievement of total score of six or more (out of 10) from individual scores, in four domains, which are given as follows:

- Number and sites of joints involved (score range 0–5 )†
- Serologic abnormality RF or ACPA auto antibody positivity (score range 0–3)
- Elevated acute-phase reactants, that is, ESR or CRP (score range 0–1)
- Duration of symptoms > 6 weeks (score range 0–1)

*Source*: Aletaha D, Neogi T, Silman AJ, et al. Rheumatoid arthritis classification criteria: An American College of Rheumatology/European League Against Rheumatism collaborative initiative. Arthritis Rheum. 2010;62:2569–2581. https://doi.org/10.1002/art.27584

†, Includes imaging evidence of synovitis.

ACR, American College of Rheumatology; EULAR, European League Against Rheumatism; RF, rheumatoid factor; ACPA, anti-citrullinated peptide antibodies; ESR, erythrocyte sedimentation rate; CRP, C-reactive protein.

Rheumatoid arthritis is associated with both articular and extra-articular manifestations. Articular involvement is classically a symmetrical inflammatory polyarthritis affecting both small and large joints. Joint disease is characterised by synovial thickening, bone oedema, bone erosions, joint space narrowing, joint subluxations and specific deformities. The small joints of the hand and feet are commonly affected in early disease. The presence of peripheral joint erosions is associated with cervical spine involvement.^6^ Changes in the cervical spine include erosion of the odontoid process, cranial settling, atlanto-axial subluxation, erosions of vertebral body margins and spinous processes, and disc space narrowing with resultant apophyseal joint ankylosis. Cranial settling and atlanto-axial subluxations pose high risk for cervical cord compression.^7^ Progression of cervical spine damage in RA creates an increased risk for myelopathy and sudden death because of spinal cord and brainstem compression.^6^

## Conventional imaging

**FIGURE 1:**
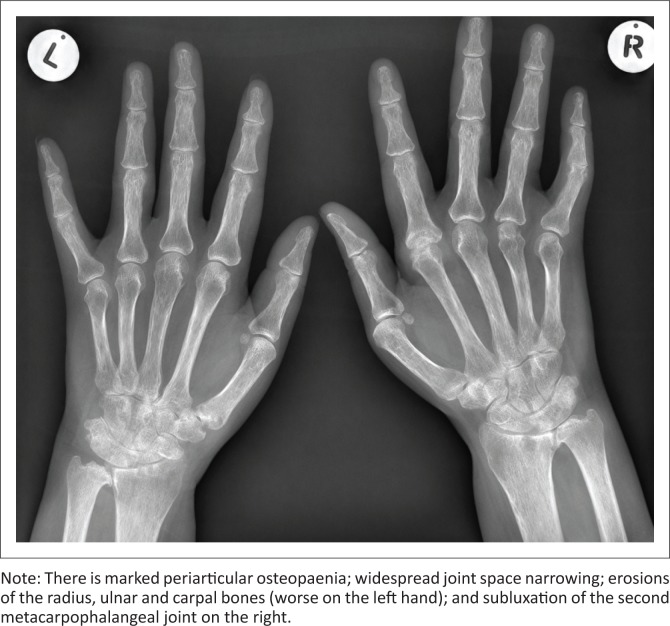
Fontal radiograph of both hands demonstrating bilateral symmetrical disease.

Conventional radiography is still used in the assessment of patients with RA. It is; however, not sensitive enough to detect changes such as bone erosions in early disease.^8,9^ It is important to remember that most of what we know today about the pathology of RA originates from plain conventional radiography. Radiography emphasises the importance of cortical bone, which is very clear on normal X-rays because of its calcium content. Erosion of cortical bone is known to be the main characteristic of erosive RA^5^ (Figure 1). Radiographs still represent a useful technique despite its limitations, because of easy availability, reliability, experience and relative low cost.^10^ Many clinical trials still use radiographic progression as an outcome measure, with radiographic scoring methods well established and sensitive to change.^11^ Disadvantages of radiographs include the following: low sensitivity to detect early joint damage, assessment of inflammatory joint involvement is indirect and insufficient because only peri-articular soft tissue swelling is detected, three-dimensional structures are shown in two dimensions and ionising radiation is used.

## Ultrasound

**FIGURE 2:**
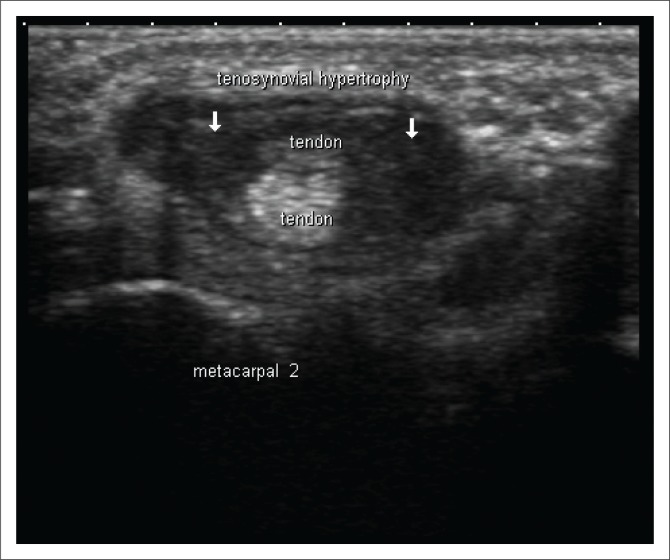
Transverse ultrasound image at the level of the second metacarpal demonstrating tenosynovitis of the extensor tendons of the hand.

**FIGURE 3:**
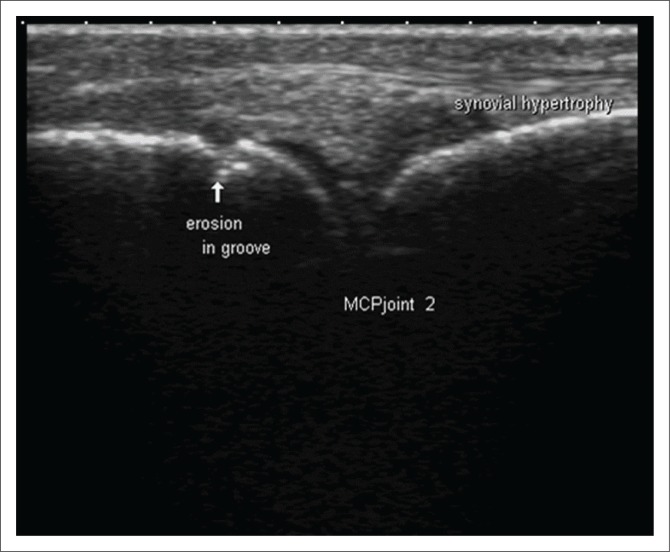
Longitudinal ultrasound at the level of the second metacarpophalangeal joint shows synovial hypertrophy with an early erosion.

Ultrasonography allows valuable assessment of soft tissues and can distinguish synovial thickening, presence of fluid in joints, bursae and tendon sheaths, basic abnormalities of tendons, ligaments, entheses and small erosions^8^ (Figure 2). High-resolution US equipment using high frequency transducers makes it possible to assess in detail the smallest anatomical alterations, which is of great value for early diagnosis and monitoring of chronic arthritis. Synovial hypertrophy is a characteristic of chronic synovitis and is regarded as a very reliable biomarker of aggressive RA. Synovial hypertrophy can be seen as circumscribed polypoid structures or have a bushy appearance on US images. Various features and the distribution of cartilage damage can be analysed in great detail with US, while bone erosions as small as one-tenth of a millimetre can be detected (Figure 3). More precise diagnosis based on the identification of specific anatomical targets can be made when US findings are combined with clinical data in patients with early disease, especially when they have seronegative RA. Up to 50% of patients with early RA do not test positive for RA-associated antibodies (RF or anti-CCP Ab).^2^ The combination of higher spatial resolution and multi-planar exploration makes US superior when compared to conventional radiography. The higher spatial resolution of US makes it possible to examine tendons in great detail, and the following can be detected: tendon sheath widening, inhomogeneity of tendon structure, localised reduction of tendon diameter, contour defects, synovial cysts, interruption, fragmentation, disappearance of echotexture and tears in the tendon.^12^ Ultrasound-guided joint and soft tissue aspirations or infiltrations allow for increased accuracy. Ultrasound has the following disadvantages: it requires additional training, is not always reproducible (examiner dependent) and is not suited for the assessment of deep joints.^10^

**FIGURE 4:**
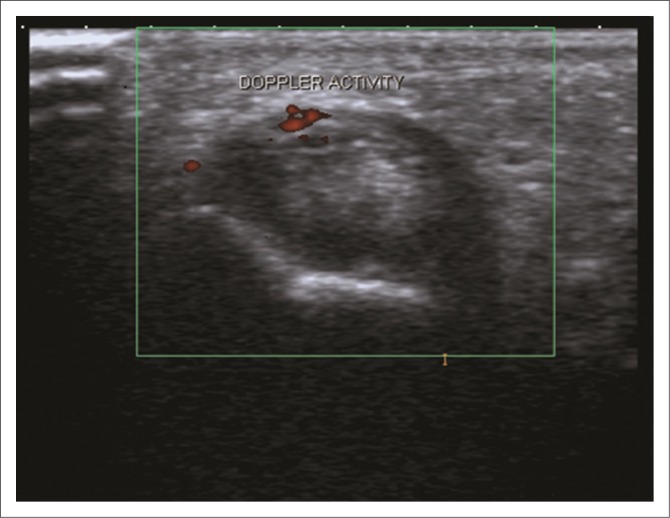
Transverse Doppler ultrasound demonstrating positive Doppler activity in the tendon sheath in keeping with active disease.

Doppler US is used to evaluate soft tissue hyperaemia,^12^ and it can be used to distinguish between active and inactive inflammatory tissue^8^ (Figure 4). Ongoing angiogenesis in areas of synovial hypertrophy is responsible for the intra-articular Doppler signal in patients with chronic arthritis. The continued presence of intensely perfused areas of synovial hypertrophy inside the joint is a reliable indicator of insufficient response to therapy and is predictive of the development of erosions.^12^

## Magnetic resonance imaging

**FIGURE 5:**
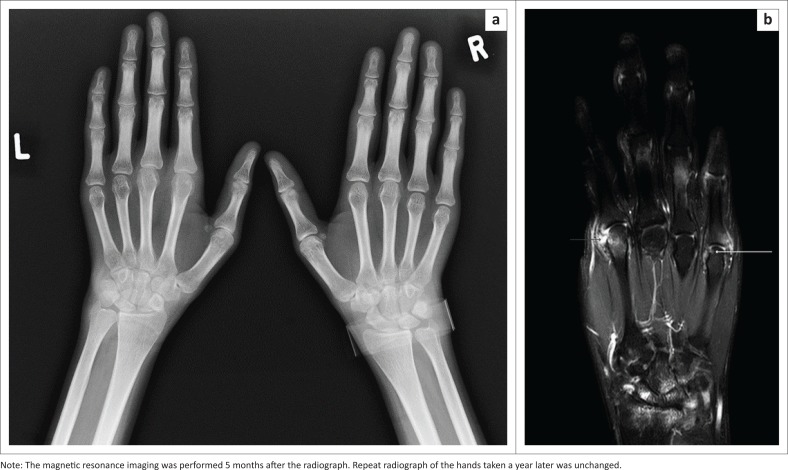
(a) Frontal radiograph of the both hands shows no evidence of erosive disease. (b) Post-contrast fat-suppressed coronal T1-weighted magnetic resonance imaging of the same hand demonstrating an erosion (white arrow) and active synovitis (grey arrow).

**FIGURE 6:**
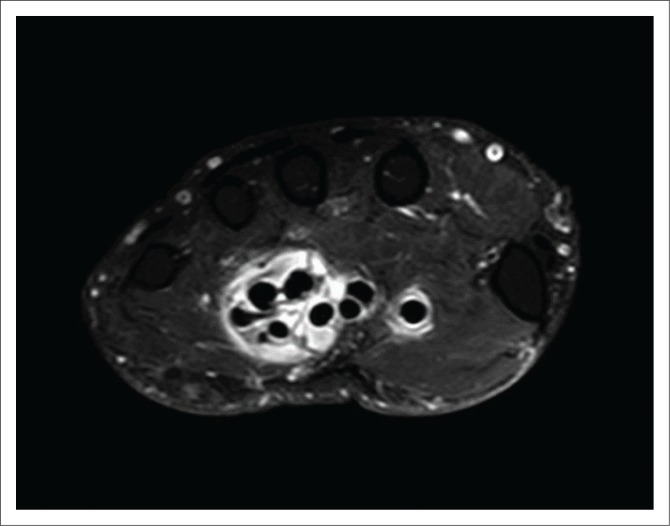
Axial proton density-weighted fat-saturated post-contrast magnetic resonance imaging at the level of the metacarpal bones demonstrating enhancement of the flexor tendons within the flexor compartment in keeping with tenosynovitis.

**FIGURE 7:**
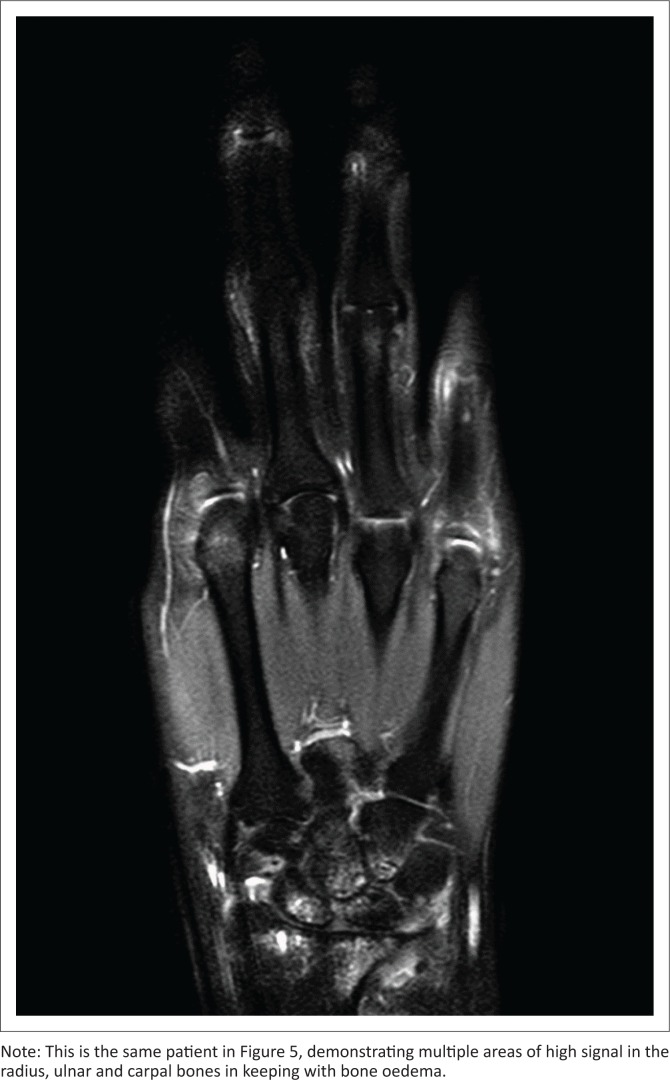
Proton density-weighted fat-saturated coronal magnetic resonance imaging.

Magnetic resonance imaging can assess all the structures affected by RA. These include soft tissue, cartilage and bones. This imaging method is highly sensitive and can detect early erosions up to three years before they may be seen with conventional radiography (Figure 5 a and b). A small dedicated extremity coil with thin slices, not > 3 mm, is advised. Magnetic resonance imaging sequences, used accordingly, are hand protocols, commonly utilised worldwide. The T1-weighted (T1W) sequence is used to detect anatomy of the imaged hand. T2-weighted (T2W), proton density-weighted fat-saturated (PDW-FS) and short-tau inversion recovery (STIR) sequences are ideal modalities to detect free fluid and regions of inflammation. In RA, this would then assist in easy diagnosis of synovitis, tenosynovitis (Figure 6), synovial effusions and bone oedema (Figure 7)^6^. Diffusion-weighted imaging (DWI) sequences, together with T2W and STIR sequences, offer feasibility in identifying synovitis in the wrist and hand, without the use of intravenous gadolinium in patients in whom contrast is contra-indicated.^13^ Active disease is demonstrated by high signal on DWI at high B values as opposed to low signal of normal bone marrow. The disadvantage of DWI is the low signal-to-noise ratio and artefacts from the inhomogeneities in the magnetic fields when used in the hands and feet.^14^

**FIGURE 8:**
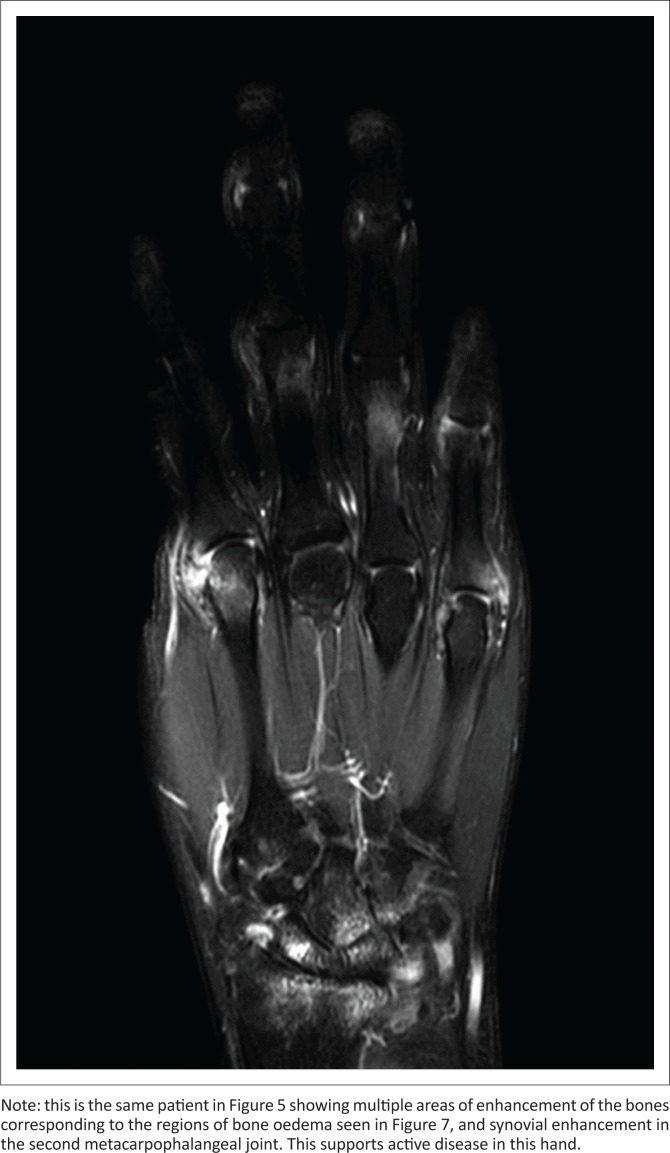
Post-contrast fat-suppressed T1-weighted coronal magnetic resonance imaging.

Gadolinium-contrasted T1W sequences allow further detection of active inflammation in areas of enhanced vascularity. Fat suppression in the post-contrast sequences allows contrast-enhanced tissues to be demonstrated more easily (Figure 8).^15^ Dynamic contrast imaging (DCE) using time-intensity curves may be employed and/or applied objectively to quantify synovial inflammation and is useful for early diagnosis and monitoring of therapy.^13^

Delayed gadolinium-enhanced MRI of cartilage is a technique developed to assess early loss of collagen and proteoglycans in cartilage before it is visible macroscopically.^16^ The functional sequence used more commonly for the assessment of cartilage however, is T2 mapping, which acquires multiple TE’s in a single sequence. High T2 correlates with increased water content and decreased collagen and proteoglycan content in cartilage, in keeping with regions of cartilage injury.^14^

Bone marrow oedema (BME) actually refers to tissue water. The high T2W signal of MRI comes from the protons in free water molecules which are found inside cells (not lipocytes) and blood vessels and are concentrated in areas where inflammation is present. Inflammatory lesions are detected by using the sensitive T2W and/or PDW sequences where inflammation is seen as a bright signal. Calcified cortical bone and trabecular bone are seen as black voids on T2W images, while the adjacent tissue, which is usually marrow fat in the normal subchondral bone, generates a signal that silhouettes the actual bone. Bone trabeculae are very small and difficult to see. Magnetic resonance imaging BME is present in many different conditions and is not disease specific, but it has a special significance in RA because it is not only an indicator of inflammation but a marker of bone pathology and future bone damage as well. Evidence from clinical studies showed that synovitis (increased synovial thickness) is greater in joints where BME is present. It also showed that treatment with the anti-TNF agent, Golimumab, decreased the CRP (which is normally associated with therapeutic response); this decrease in CRP runs parallel with reductions in synovitis and BME. These measures correlate strongly with each other and are often all found in the same joint; but evidence from further studies showed that the absence of BME made the formation of MRI erosions highly unlikely over a period of 12 months. In the presence of BME; however, the likelihood that erosions would form was drastically increased. Various groups in different studies have shown that BME is the strongest of conventional and imaging biomarkers for the prediction of erosive progression of RA.^5^

## Discussion

Modern treatment of the disease requires very early detection and rigid control of inflammatory arthritis. Magnetic resonance imaging has increased sensitivity for detecting RA pathology over clinical examination and radiographs, and it can be used for the benefit of patients suffering from this disease. The exact role of MRI in managing RA patients in clinical practice requires further research, especially regarding the determination of clinical algorithms for the use of MRI, the role of MRI imaging in monitoring existing RA and the understanding of how imaging can help to improve the cost-effectiveness of current biologic treatment regimens used in the management of RA.^17^

Evidence from different clinical studies has shown that conventional radiography, US or MRI can be used to confirm the diagnosis when clinical and laboratory data on their own are not enough. MRI BME is a strong predictor of bone damage and can be used as a prognostic indicator in RA. Inflammation detected by imaging may be a more accurate reflection of therapeutic response than the clinical measures used to monitor disease activity. MRI and US can be used to monitor disease progression in patients with RA.^8,9^ Clinical trials evaluating expensive therapies could use MRI as an outcome measure allowing for shorter trials as MRI changes are apparently much faster.^4^

In 2003, the Outcome Measures in Rheumatoid Arthritis Clinical Trials (OMERACT) group with the Rheumatoid Arthritis Magnetic Resonance Imaging Score (RAMRIS) system established a highly reliable sum-score based on semi-quantitative rating of severity of synovitis, bone oedema, joint inflammation and erosions in the hands and wrist joints.^6,15^

According to the OMERACT group, synovitis is an area in the synovial compartment that shows above-normal enhancement after gadolinium contrast administration of a thickness greater than the width of the normal synovium. Erosions are sharply delineated bone lesions that are located at the joint margins.^3^

The RAMRIS system has been shown to be a useful, sensitive tool for the evaluation of therapy response in RA. Rheumatoid Arthritis Magnetic Resonance Imaging Score criteria use a sum-score of 23 joint sites of the hand, comprising metacarpophalangeal (MCP) joints two–five, carpo-metacarpophalangeal (CMC) joints one–five, intercarpal joints, radiocarpal joints and radioulnar joints; yielding the sum of individual joints subscore for synovitis, BME and erosions.^18^

Schleich et al.^18^ conducted a study in January 2015 to evaluate inflammation and joint destruction of the dominant hand in patients with RA, using the modified RAMRIS 5. This study was performed in Germany. Patients had MRI scans at baseline and also had follow-up scans performed accordingly. On both occasions, 23 joints of the hands and five joints of the hand, respectively, were scored, and a comparison study was performed. Assessment of the hands was performed using the RAMRIS and RAMRIS 5 simultaneously and graded according to bone oedema, erosions and synovitis. Joints used in RAMRIS 5 include MCP 2 and 3, capitate bone, triquetral bone and distal ulna for analysis of erosions and bone oedema. These are joints and bones that are commonly affected in RA.

Schleich et al. found that RAMRIS 5 can be used in assessing inflammatory joint changes and therapy monitoring in patients affected with RA. Rheumatoid Arthritis Magnetic Resonance Imaging Score 5 has been found to be a time-and resource-saving technique and may have a role in routine clinical practice.^18^

Studies have shown both MRI and US to be highly sensitive in assessing the inflammation of joints.^19,20^ US; however, cannot image for BME, a strong indicator of future bone damage and disease progression.^5^ It may also fail to adequately assess some joint regions^11^ and is extremely operator dependent but has the advantage of being cheaper and more readily available and easily allows for intervention such as US-guided intra-articular injections. Magnetic resonance imaging has the advantage of greater joint coverage and the detection of BME but is more expensive and less accessible in the resource-constrained environment. Magnetic resonance imaging, therefore, has an important role to play in early diagnosis of RA, and patients may then be followed up by US for monitoring and treatment response, provided an experienced operator is available. This would prove cheaper, safer and more accessible. However, if US is equivocal or cannot reach the region of interest, then MRI is advised.^21^ Further research is needed to optimise the roles of these advanced imaging modalities in RA to provide cost-effective management of patients.^16^

## Conclusion

Conventional radiography has been the gold standard for imaging in RA for a long time, but the sensitivity for structural damage in the diagnosis of RA is low and disease activity cannot be assessed. Despite these limitations, it remains a useful modality in routine clinical management of patients with RA. US and MRI (especially contrast-enhanced MRI) are rapidly becoming the imaging examinations of choice for the detection of early disease in patients because of increased sensitivity. Newer imaging applications are useful to diagnose and monitor disease progression in RA in routine clinical practice. In resource-poor countries, these applications could potentially assist earlier diagnosis, when patients are more likely to respond to conventional therapies and could also help stratify subgroups of patients most likely to respond to expensive biologic therapies.
